# *catena*-Poly[[diaqua­calcium]bis­[μ-2-(1,3-dioxoisoindolin-2-yl)acetato]-κ^3^ *O*,*O*′:*O*;κ^3^ *O*:*O*,*O*′]

**DOI:** 10.1107/S1600536811027851

**Published:** 2011-07-16

**Authors:** Moazzam H. Bhatti, Uzma Yunus, Sohail Saeed, Syed Raza Shah, Wing-Tak Wong

**Affiliations:** a Department of Chemistry, Research Complex, Allama Iqbal Open University, Islamabad 44000, Pakistan; b Department of Chemistry, The University of Hong Kong, Pokfulam Road, Pokfulam, Hong Kong SAR, People’s Republic of China

## Abstract

In the title complex, [Ca(C_10_H_6_NO_4_)_2_(H_2_O)_2_]_*n*_, the Ca^II^ atom lies on a twofold rotation axis and adopts a dodeca­hedral geometry. The Ca^II^ atom is octa­coordinated by two O atoms from two water mol­ecules and six O atoms from four acetate ligands. Each acetate acts as a tridentate ligand bridging two Ca^II^ atoms, resulting in a chain running along the *c* axis. O—H⋯O and C—H⋯O hydrogen bonds connect the chains into a two-dimensional network parallel to [011]. π–π inter­actions between adjacent isoindoline-1,3-dione rings [centroid–centroid distance = 3.4096 (11) Å] further consolidate the structure. One of the carboxylate O atoms is disordered over two sites in a 0.879 (12):0.121 (12) ratio.

## Related literature

For background to *N*-phthaloylglycine, see: Khan & Ismail (2002 ▶). For related structures, see: Barooah *et al.* (2006 ▶).
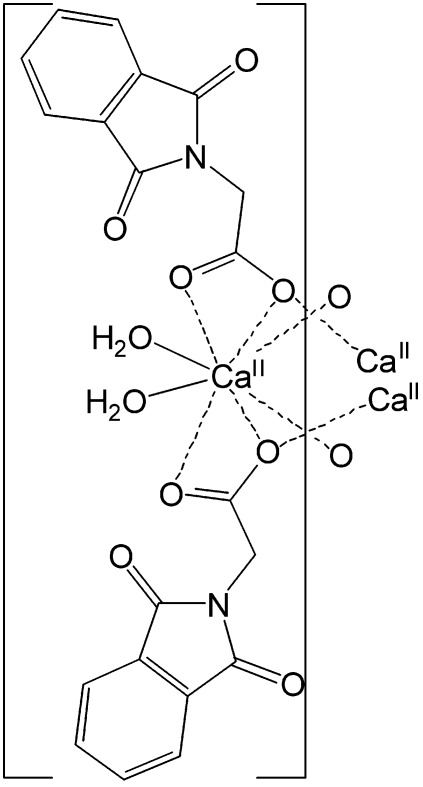


## Experimental

### 

#### Crystal data

[Ca(C_10_H_6_NO_4_)_2_(H_2_O)_2_]
*M* *_r_* = 484.43
Monoclinic, 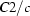

*a* = 32.752 (1) Å
*b* = 9.0435 (3) Å
*c* = 6.9753 (3) Å
β = 99.020 (2)°
*V* = 2040.48 (13) Å^3^
*Z* = 4
Mo *K*α radiation
μ = 0.37 mm^−1^
*T* = 296 K
0.34 × 0.32 × 0.32 mm

#### Data collection

Bruker APEXII CCD diffractometer
Absorption correction: multi-scan (*SADABS*; Sheldrick, 2008*a* ▶) *T*_min_ = 0.884, *T*_max_ = 0.890
12481 measured reflections
2339 independent reflections
1847 reflections with *I* > 2σ(*I*)
*R*_int_ = 0.042

#### Refinement

*R*[*F* ^2^ > 2σ(*F* ^2^)] = 0.036
*wR*(*F* ^2^) = 0.102
*S* = 1.04
2339 reflections
160 parameters
3 restraints
H atoms treated by a mixture of independent and constrained refinement
Δρ_max_ = 0.28 e Å^−3^
Δρ_min_ = −0.23 e Å^−3^

### 

Data collection: *APEX2* (Bruker, 2007 ▶); cell refinement: *SAINT* (Bruker, 2007 ▶); data reduction: *SAINT*; program(s) used to solve structure: *SHELXS97* (Sheldrick, 2008*b* ▶); program(s) used to refine structure: *SHELXL97* (Sheldrick, 2008*b* ▶); molecular graphics: *Mercury* (Macrae *et al.*, 2008 ▶); software used to prepare material for publication: *SHELXL97*.

## Supplementary Material

Crystal structure: contains datablock(s) global, I. DOI: 10.1107/S1600536811027851/pv2422sup1.cif

Structure factors: contains datablock(s) I. DOI: 10.1107/S1600536811027851/pv2422Isup2.hkl

Additional supplementary materials:  crystallographic information; 3D view; checkCIF report

## Figures and Tables

**Table 1:** Hydrogen-bond geometry (Å, °)

*D*—H⋯*A*	*D*—H	H⋯*A*	*D*⋯*A*	*D*—H⋯*A*
O5—H5*A*⋯O4^i^	0.82 (1)	2.10 (1)	2.907 (2)	171 (3)
O5—H5*B*⋯O4^ii^	0.82 (1)	2.49 (2)	3.095 (2)	131 (2)
C8—H8⋯O2^iii^	0.93	2.47	3.318 (2)	151

Symmetry codes: (i) 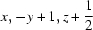; (ii) 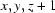; (iii) 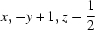.

**Fig. 1.**
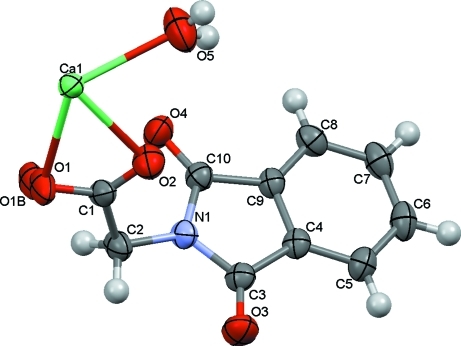
The asymmetric unit of the title complex drwan with 50% probability thermal ellipsoids showing the atom numbering scheme.

**Fig. 2.**
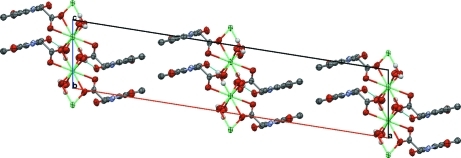
The packing diagram of the complex projected down the b axis showing the 1-D chain running parallel to the c axis; the cyan dotted lines indicate the H-bonding interactions.

## supplementary crystallographic information

### Comment

N-Phthaloylglycine is a simple *N*-phthaloylamino acid which has been widely studied for cleavage with various amines (Khan & Ismail, 2002) and metal complexation with interesting supramolecular structures (Barooah *et al.*, 2006). In an attempt to synthesis calcium(II) complex of N-phthaloylglycine, we have prepared a calcium complex of N-phthaloylglycine as the title compound and studied it crystal structure which is presented in this article.

In the title complex, the calcium ayom is octa-coordinated to two oxygen atoms from two water solvates and to 6 oxygen atoms from four acetate ligands. Each acetate acts as a tridentate ligand bridging two calcium centres resulting in a 1-D polymeric chain running along the *c*-axis. The calcium atom sits on a 2-fold axis, thus the asymmetric unit contains only half of the complex (Fig. 1).

The oxygen atom O1 is slightly disordered over two sites with occupancy factors 0.879 (12) and 0.121 (12). The acetate ring plane, C1/C2/O1/O2, makes a dihedral angle of 75.62 (8)° (77.0 (4)° for C1/C2/O1B/O2) with the ring plane of the isoindole-1,3-dione, N1/O3/O4/C3—C10.

There are inter-molecular O—H···O H-bonding interactions which link the molecules into a 2-D network parallel to the [0 1 1] plane (Fig. 2). There are also weak π–π interactions between adjacent isoindole-1,3-dione rings along the *c* axis in the crystal lattice; the distance between the centroids of the rings C4—C9 and (N1/C3/C4/C9/C10)^*^ (^*^: *x*, 1 - *y*, 1/2 + *z*) being 3.4096 (11) Å. These π–π interactions help stacking the acetate ligand plane along the *c*-axis in the lattice.

### Experimental

The title compound was prepared from the reaction of CaCl_2_.2H_2_O (0.01 mol) and sodium (1,3-dioxo-1,3-dihydro-2*H*-isoindol-2-yl)acetate (0.02 mol) solution. Sodium (1,3-dioxo-1,3-dihydro-2*H*-isoindol-2-yl)acetate was first obtained by adding 0.02 mol of (1,3-dioxo-1,3-dihydro-2*H*-isoindol-2-yl)acetic acid to an aqueous solution of 0.02 mol NaHCO_3_. The mixture was set aside to crystallize at ambient temperature for several days, giving suitable colorless single crystals.

### Refinement

All of the C-bound H atoms were observable from difference Fourier map but were placed at geometrical positions with C—H = 0.93 and 0.97 Å for phenyl and methylene H-atoms, respectively, and were refined using riding model with *U*_iso_(H) = 1.2*U*_eq_(C). The O-bound H-atoms were located from difference Fourier map and refined with bond distance restrains O–H = 0.82 (1) Å and H···H = 1.32 (1) Å with the thermal parameters set at *U*_iso_(H) = 1.5*U*_eq_(O). The oxygen atom O1 was disordered over two sites, with site occupancy factors 0.879 (12) and 0.121 (12).

### Crystal data

**Table:**

[Ca(C_10_H_6_NO_4_)_2_(H_2_O)_2_]	*F*(000) = 1000
*M**_r_* = 484.43	*D*_x_ = 1.577 Mg m^−^^3^
Monoclinic, *C*2/*c*	Mo *K*α radiation, λ = 0.71073 Å
Hall symbol: -C 2yc	Cell parameters from 12481 reflections
*a* = 32.752 (1) Å	θ = 2.3–27.5°
*b* = 9.0435 (3) Å	µ = 0.37 mm^−^^1^
*c* = 6.9753 (3) Å	*T* = 296 K
β = 99.020 (2)°	Block, colourless
*V* = 2040.48 (13) Å^3^	0.34 × 0.32 × 0.32 mm
*Z* = 4	

### Data collection

**Table:**

Bruker APEXII CCD diffractometer	2339 independent reflections
Radiation source: fine-focus sealed tube	1847 reflections with *I* > 2σ(*I*)
graphite	*R*_int_ = 0.042
ω and φ scans	θ_max_ = 27.5°, θ_min_ = 2.3°
Absorption correction: multi-scan (*SADABS*; Sheldrick, 2008*a*)	*h* = −42→42
*T*_min_ = 0.884, *T*_max_ = 0.890	*k* = −11→11
12481 measured reflections	*l* = −8→9

### Refinement

**Table:**

Refinement on *F*^2^	Primary atom site location: structure-invariant direct methods
Least-squares matrix: full	Secondary atom site location: difference Fourier map
*R*[*F*^2^ > 2σ(*F*^2^)] = 0.036	Hydrogen site location: inferred from neighbouring sites
*wR*(*F*^2^) = 0.102	H atoms treated by a mixture of independent and constrained refinement
*S* = 1.04	*w* = 1/[σ^2^(*F*_o_^2^) + (0.0543*P*)^2^ + 0.9315*P*] where *P* = (*F*_o_^2^ + 2*F*_c_^2^)/3
2339 reflections	(Δ/σ)_max_ = 0.001
160 parameters	Δρ_max_ = 0.28 e Å^−^^3^
3 restraints	Δρ_min_ = −0.23 e Å^−^^3^

### Special details

**Table:**

Geometry. All e.s.d.'s (except the e.s.d. in the dihedral angle between two l.s. planes) are estimated using the full covariance matrix. The cell e.s.d.'s are taken into account individually in the estimation of e.s.d.'s in distances, angles and torsion angles; correlations between e.s.d.'s in cell parameters are only used when they are defined by crystal symmetry. An approximate (isotropic) treatment of cell e.s.d.'s is used for estimating e.s.d.'s involving l.s. planes.
Refinement. Refinement of *F*^2^ against ALL reflections. The weighted *R*-factor *wR* and goodness of fit *S* are based on *F*^2^, conventional *R*-factors *R* are based on *F*, with *F* set to zero for negative *F*^2^. The threshold expression of *F*^2^ > σ(*F*^2^) is used only for calculating *R*-factors(gt) *etc*. and is not relevant to the choice of reflections for refinement. *R*-factors based on *F*^2^ are statistically about twice as large as those based on *F*, and *R*- factors based on ALL data will be even larger.

### Fractional atomic coordinates and isotropic or equivalent isotropic displacement parameters (Å^2^)

**Table:**

	*x*	*y*	*z*	*U*_iso_*/*U*_eq_	Occ. (<1)
Ca1	0.0000	0.12307 (5)	0.2500	0.02944 (16)	
O1	0.03670 (7)	0.0467 (4)	−0.0362 (7)	0.0520 (9)	0.879 (12)
O1B	0.0322 (5)	0.084 (2)	−0.126 (5)	0.0520 (9)	0.121 (12)
O2	0.07083 (4)	0.19128 (16)	0.1763 (2)	0.0468 (4)	
O3	0.18731 (4)	0.15606 (15)	0.0563 (2)	0.0509 (4)	
O4	0.07509 (4)	0.44337 (16)	−0.1576 (2)	0.0471 (4)	
O5	0.02298 (6)	0.3258 (2)	0.4645 (3)	0.0691 (5)	
H5A	0.0399 (7)	0.384 (3)	0.433 (4)	0.104*	
H5B	0.0228 (10)	0.337 (4)	0.5810 (11)	0.104*	
N1	0.12562 (4)	0.27067 (16)	−0.0647 (2)	0.0338 (3)	
C1	0.06666 (5)	0.12894 (18)	0.0183 (3)	0.0341 (4)	
C2	0.09973 (6)	0.1420 (2)	−0.1122 (3)	0.0387 (4)	
H2A	0.1168	0.0539	−0.0985	0.046*	
H2B	0.0865	0.1484	−0.2465	0.046*	
C3	0.16724 (5)	0.2673 (2)	0.0189 (3)	0.0343 (4)	
C4	0.17993 (5)	0.4252 (2)	0.0462 (3)	0.0320 (4)	
C5	0.21772 (6)	0.4866 (2)	0.1170 (3)	0.0391 (4)	
H5	0.2407	0.4280	0.1583	0.047*	
C6	0.22000 (6)	0.6402 (2)	0.1243 (3)	0.0438 (5)	
H6	0.2450	0.6852	0.1737	0.053*	
C7	0.18598 (6)	0.7274 (2)	0.0598 (3)	0.0436 (5)	
H7	0.1886	0.8298	0.0661	0.052*	
C8	0.14793 (6)	0.6653 (2)	−0.0144 (3)	0.0380 (4)	
H8	0.1251	0.7237	−0.0596	0.046*	
C9	0.14567 (5)	0.5126 (2)	−0.0178 (2)	0.0311 (4)	
C10	0.11039 (5)	0.41339 (19)	−0.0884 (3)	0.0331 (4)	

### Atomic displacement parameters (Å^2^)

**Table:**

	*U*^11^	*U*^22^	*U*^33^	*U*^12^	*U*^13^	*U*^23^
Ca1	0.0250 (2)	0.0241 (2)	0.0391 (3)	0.000	0.00463 (19)	0.000
O1	0.0402 (9)	0.0575 (14)	0.062 (2)	−0.0247 (9)	0.0201 (11)	−0.0249 (15)
O1B	0.0402 (9)	0.0575 (14)	0.062 (2)	−0.0247 (9)	0.0201 (11)	−0.0249 (15)
O2	0.0444 (8)	0.0546 (9)	0.0438 (8)	−0.0091 (7)	0.0146 (6)	−0.0066 (7)
O3	0.0449 (8)	0.0355 (7)	0.0711 (11)	0.0061 (6)	0.0050 (7)	0.0046 (7)
O4	0.0340 (7)	0.0455 (8)	0.0583 (10)	−0.0011 (6)	−0.0033 (6)	0.0005 (7)
O5	0.0810 (13)	0.0671 (11)	0.0618 (11)	−0.0256 (9)	0.0189 (10)	−0.0254 (10)
N1	0.0300 (7)	0.0278 (7)	0.0450 (9)	−0.0056 (6)	0.0102 (6)	−0.0018 (6)
C1	0.0289 (8)	0.0235 (8)	0.0510 (12)	−0.0035 (6)	0.0093 (8)	−0.0023 (8)
C2	0.0388 (10)	0.0311 (9)	0.0486 (11)	−0.0100 (7)	0.0138 (8)	−0.0081 (8)
C3	0.0325 (9)	0.0339 (9)	0.0379 (10)	−0.0019 (7)	0.0102 (8)	0.0029 (8)
C4	0.0327 (9)	0.0353 (9)	0.0292 (9)	−0.0053 (7)	0.0082 (7)	0.0017 (7)
C5	0.0344 (9)	0.0444 (10)	0.0376 (10)	−0.0056 (8)	0.0033 (8)	0.0028 (9)
C6	0.0440 (11)	0.0475 (11)	0.0396 (11)	−0.0193 (9)	0.0052 (9)	−0.0022 (9)
C7	0.0582 (12)	0.0322 (9)	0.0413 (11)	−0.0149 (9)	0.0107 (10)	−0.0021 (8)
C8	0.0477 (11)	0.0311 (9)	0.0357 (10)	−0.0010 (8)	0.0076 (8)	0.0024 (8)
C9	0.0334 (9)	0.0322 (9)	0.0283 (9)	−0.0058 (7)	0.0069 (7)	0.0000 (7)
C10	0.0329 (9)	0.0326 (9)	0.0344 (10)	−0.0032 (7)	0.0072 (7)	0.0005 (7)

### Geometric parameters (Å, °)

**Table:**

Ca1—O1B^i^	2.258 (15)	O5—H5B	0.8199 (10)
Ca1—O1B^ii^	2.258 (15)	N1—C10	1.384 (2)
Ca1—O1^i^	2.338 (2)	N1—C3	1.396 (2)
Ca1—O1^ii^	2.338 (2)	N1—C2	1.447 (2)
Ca1—O5	2.4111 (16)	C1—C2	1.525 (3)
Ca1—O5^iii^	2.4111 (16)	C2—H2A	0.9700
Ca1—O2	2.5298 (13)	C2—H2B	0.9700
Ca1—O2^iii^	2.5298 (13)	C3—C4	1.492 (2)
Ca1—O1^iii^	2.581 (3)	C4—C5	1.375 (2)
Ca1—O1	2.581 (3)	C4—C9	1.388 (2)
Ca1—O1B	2.99 (3)	C5—C6	1.391 (3)
Ca1—O1B^iii^	2.99 (3)	C5—H5	0.9300
O1—C1	1.242 (2)	C6—C7	1.382 (3)
O1—Ca1^i^	2.338 (2)	C6—H6	0.9300
O1B—C1	1.45 (2)	C7—C8	1.391 (3)
O1B—Ca1^i^	2.258 (15)	C7—H7	0.9300
O2—C1	1.226 (2)	C8—C9	1.383 (3)
O3—C3	1.207 (2)	C8—H8	0.9300
O4—C10	1.212 (2)	C9—C10	1.485 (2)
O5—H5A	0.8200 (10)		
			
O1B^i^—Ca1—O1B^ii^	67.6 (16)	O5—Ca1—O1B^iii^	70.4 (6)
O1B^i^—Ca1—O1^i^	17.6 (8)	O5^iii^—Ca1—O1B^iii^	120.9 (3)
O1B^ii^—Ca1—O1^i^	82.0 (9)	O2—Ca1—O1B^iii^	131.8 (5)
O1B^i^—Ca1—O1^ii^	82.0 (9)	O2^iii^—Ca1—O1B^iii^	52.5 (3)
O1B^ii^—Ca1—O1^ii^	17.6 (8)	O1^iii^—Ca1—O1B^iii^	11.8 (5)
O1^i^—Ca1—O1^ii^	97.9 (3)	O1—Ca1—O1B^iii^	156.4 (4)
O1B^i^—Ca1—O5	162.2 (7)	O1B—Ca1—O1B^iii^	166.6 (9)
O1B^ii^—Ca1—O5	108.2 (9)	C1—O1—Ca1^i^	153.0 (2)
O1^i^—Ca1—O5	167.07 (8)	C1—O1—Ca1	92.55 (18)
O1^ii^—Ca1—O5	91.40 (14)	Ca1^i^—O1—Ca1	114.46 (8)
O1B^i^—Ca1—O5^iii^	108.2 (9)	C1—O1B—Ca1^i^	139.9 (19)
O1B^ii^—Ca1—O5^iii^	162.2 (7)	C1—O1B—Ca1	72.8 (11)
O1^i^—Ca1—O5^iii^	91.40 (14)	Ca1^i^—O1B—Ca1	103.1 (9)
O1^ii^—Ca1—O5^iii^	167.07 (8)	C1—O2—Ca1	95.40 (11)
O5—Ca1—O5^iii^	80.99 (11)	Ca1—O5—H5A	119 (2)
O1B^i^—Ca1—O2	120.8 (5)	Ca1—O5—H5B	131 (2)
O1B^ii^—Ca1—O2	83.9 (4)	H5A—O5—H5B	107 (3)
O1^i^—Ca1—O2	115.25 (7)	C10—N1—C3	112.36 (14)
O1^ii^—Ca1—O2	83.90 (6)	C10—N1—C2	122.35 (15)
O5—Ca1—O2	74.56 (6)	C3—N1—C2	125.20 (15)
O5^iii^—Ca1—O2	84.00 (6)	O2—C1—O1	121.4 (2)
O1B^i^—Ca1—O2^iii^	83.9 (4)	O2—C1—O1B	135.8 (8)
O1B^ii^—Ca1—O2^iii^	120.8 (5)	O2—C1—C2	120.75 (15)
O1^i^—Ca1—O2^iii^	83.90 (6)	O1—C1—C2	117.7 (2)
O1^ii^—Ca1—O2^iii^	115.25 (7)	O1B—C1—C2	99.1 (10)
O5—Ca1—O2^iii^	84.00 (6)	O2—C1—Ca1	59.82 (10)
O5^iii^—Ca1—O2^iii^	74.56 (6)	O1—C1—Ca1	62.24 (17)
O2—Ca1—O2^iii^	151.77 (7)	O1B—C1—Ca1	78.9 (9)
O1B^i^—Ca1—O1^iii^	80.2 (6)	C2—C1—Ca1	175.44 (13)
O1B^ii^—Ca1—O1^iii^	74.1 (6)	N1—C2—C1	111.80 (15)
O1^i^—Ca1—O1^iii^	93.58 (5)	N1—C2—H2A	109.3
O1^ii^—Ca1—O1^iii^	65.54 (8)	C1—C2—H2A	109.3
O5—Ca1—O1^iii^	82.03 (12)	N1—C2—H2B	109.3
O5^iii^—Ca1—O1^iii^	123.06 (6)	C1—C2—H2B	109.3
O2—Ca1—O1^iii^	140.87 (9)	H2A—C2—H2B	107.9
O2^iii^—Ca1—O1^iii^	49.82 (5)	O3—C3—N1	124.85 (17)
O1B^i^—Ca1—O1	74.1 (6)	O3—C3—C4	129.69 (17)
O1B^ii^—Ca1—O1	80.2 (6)	N1—C3—C4	105.46 (14)
O1^i^—Ca1—O1	65.54 (8)	C5—C4—C9	121.49 (17)
O1^ii^—Ca1—O1	93.58 (5)	C5—C4—C3	130.51 (17)
O5—Ca1—O1	123.06 (6)	C9—C4—C3	107.99 (14)
O5^iii^—Ca1—O1	82.03 (12)	C4—C5—C6	117.14 (18)
O2—Ca1—O1	49.82 (5)	C4—C5—H5	121.4
O2^iii^—Ca1—O1	140.87 (9)	C6—C5—H5	121.4
O1^iii^—Ca1—O1	148.96 (18)	C7—C6—C5	121.47 (17)
O1B^i^—Ca1—O1B	76.9 (9)	C7—C6—H6	119.3
O1B^ii^—Ca1—O1B	91.8 (5)	C5—C6—H6	119.3
O1^i^—Ca1—O1B	65.2 (3)	C6—C7—C8	121.36 (18)
O1^ii^—Ca1—O1B	105.4 (5)	C6—C7—H7	119.3
O5—Ca1—O1B	120.9 (3)	C8—C7—H7	119.3
O5^iii^—Ca1—O1B	70.4 (6)	C9—C8—C7	116.88 (18)
O2—Ca1—O1B	52.5 (3)	C9—C8—H8	121.6
O2^iii^—Ca1—O1B	131.8 (5)	C7—C8—H8	121.6
O1^iii^—Ca1—O1B	156.4 (4)	C8—C9—C4	121.64 (16)
O1—Ca1—O1B	11.8 (5)	C8—C9—C10	130.22 (17)
O1B^i^—Ca1—O1B^iii^	91.8 (5)	C4—C9—C10	108.11 (15)
O1B^ii^—Ca1—O1B^iii^	76.9 (9)	O4—C10—N1	124.03 (16)
O1^i^—Ca1—O1B^iii^	105.4 (5)	O4—C10—C9	129.90 (17)
O1^ii^—Ca1—O1B^iii^	65.2 (3)	N1—C10—C9	106.06 (14)

Symmetry codes: (i) −*x*, −*y*, −*z*; (ii) *x*, −*y*, *z*+1/2; (iii) −*x*, *y*, −*z*+1/2.

### Hydrogen-bond geometry (Å, °)

**Table:**

*D*—H···*A*	*D*—H	H···*A*	*D*···*A*	*D*—H···*A*
O5—H5A···O4^iv^	0.82 (1)	2.10 (1)	2.907 (2)	171 (3)
O5—H5B···O4^v^	0.82 (1)	2.49 (2)	3.095 (2)	131 (2)
C8—H8···O2^vi^	0.93	2.47	3.318 (2)	151

Symmetry codes: (iv) *x*, −*y*+1, *z*+1/2; (v) *x*, *y*, *z*+1; (vi) *x*, −*y*+1, *z*−1/2.
